# The Cytogenomic “Theory of Everything”: Chromohelkosis May Underlie Chromosomal Instability and Mosaicism in Disease and Aging

**DOI:** 10.3390/ijms21218328

**Published:** 2020-11-06

**Authors:** Ivan Y. Iourov, Svetlana G. Vorsanova, Yuri B. Yurov, Maria A. Zelenova, Oxana S. Kurinnaia, Kirill S. Vasin, Sergei I. Kutsev

**Affiliations:** 1 Mental Health Research Center, 117152 Moscow, Russia; svorsanova@mail.ru (S.G.V.); y_yurov@yahoo.com (Y.B.Y.); maria_zelenova@yahoo.com (M.A.Z.); kurinnaiaos@mail.ru (O.S.K.); vasin-ks@rambler.ru (K.S.V.); 2 Veltischev Research and Clinical Institute for Pediatrics of the Pirogov Russian National Research Medical University, Ministry of Health of Russian Federation, 125412 Moscow, Russia; 3 Department of Medical Biological Disciplines, Belgorod State University, 308015 Belgorod, Russia; 4 Research Centre for Medical Genetics, 115522 Moscow, Russia; kutsev@mail.ru

**Keywords:** chromosome, copy number variations, chromosome instability, chromosomal mosaicism, chromosomal imbalances, aneuploidy, disease, aging, pathways, cytogenomics

## Abstract

Mechanisms for somatic chromosomal mosaicism (SCM) and chromosomal instability (CIN) are not completely understood. During molecular karyotyping and bioinformatic analyses of children with neurodevelopmental disorders and congenital malformations (*n* = 612), we observed colocalization of regular chromosomal imbalances or copy number variations (CNV) with mosaic ones (*n* = 47 or 7.7%). Analyzing molecular karyotyping data and pathways affected by CNV burdens, we proposed a mechanism for SCM/CIN, which had been designated as “chromohelkosis” (from the Greek words chromosome ulceration/open wound). Briefly, structural chromosomal imbalances are likely to cause local instability (“wreckage”) at the breakpoints, which results either in partial/whole chromosome loss (e.g., aneuploidy) or elongation of duplicated regions. Accordingly, a function for classical/alpha satellite DNA (protection from the wreckage towards the centromere) has been hypothesized. Since SCM and CIN are ubiquitously involved in development, homeostasis and disease (e.g., prenatal development, cancer, brain diseases, aging), we have metaphorically (ironically) designate the system explaining chromohelkosis contribution to SCM/CIN as the cytogenomic “theory of everything”, similar to the homonymous theory in physics inasmuch as it might explain numerous phenomena in chromosome biology. Recognizing possible empirical and theoretical weaknesses of this “theory”, we nevertheless believe that studies of chromohelkosis-like processes are required to understand structural variability and flexibility of the genome.

## 1. Introduction

Despite the fact that mechanisms of chromosomal instability (CIN) and somatic chromosomal mosaicism (SCM) remain to be further explored [1,2,3], CIN-associated genome behavior (chromothripsis, chromoanasynthesis, chromoanagenesis) has already been described [4,5,6]. Uncovering underlying processes of the commonest types of SCM/CIN (e.g., aneuploidy) is generally less successful [7,8,9]. Still, somatic aneuploidy (CIN manifested as aneuploidy) has been found to result from alterations to a number of molecular/cellular pathways, gene mutations [10,11], and/or genetic–environmental interactions [12]. In the postgenomic context, it appears important to evaluate the contribution of heritable/sporadic cytogenomic variations (i.e., genomic variations at chromosomal and subchromosomal levels) to the formation of CIN and SCM using whole-genome analyses and systems biology approaches. Currently, studies of pathways from regular chromosomal imbalances and/or copy number variations (CNV) to CIN/SCM are rare.

Genome architecture at the DNA sequence level has long been recognized as playing a key role in formation of structural genome variations [13,14]. A large series of studies using a panel of molecular, cytogenomic, and bioinformatic techniques have shown that structural genomic variants (chromosomal rearrangements and CNV) frequently occur through mechanisms involving repeat sequences at the breakpoints as well as DNA recombination-based and replication-based processes [15,16,17,18,19]. At the chromosomal/subchromosomal levels, related phenomena have not been systematically addressed. However, chromosome segregation errors have been indicated to form a wide spectrum of somatic genome rearrangements [20]. These results promise the success of forthcoming studies of interplays between genome behavior at chromosomal (subchromosomal) level, CIN and SCM. Therefore, whole-genome and bioinformatic analyses of co-occurring non-mosaic and mosaic chromosomal (subchromosomal) variations or CIN in an individual may help to uncover previously unrecognized mechanisms for somatic genome variations at the chromosomal level.

In the present contribution, we describe an SNP (single nucleotide polymorphism)-array study of colocalized regular/mosaic chromosome imbalances in a cohort of children with neurodevelopmental disorders and congenital malformations. Analysis of the structural genomic variations allowed us to propose a previously unknown pathway from regular chromosomal imbalances or CNV to SCM (CIN). Furthermore, we have introduced a kind of theory, which might be relevant to numerous areas of chromosome research, cytogenomics (molecular cytogenetics), and medical cytogenetics.

## 2. Results and Discussion

**Figure 1:**
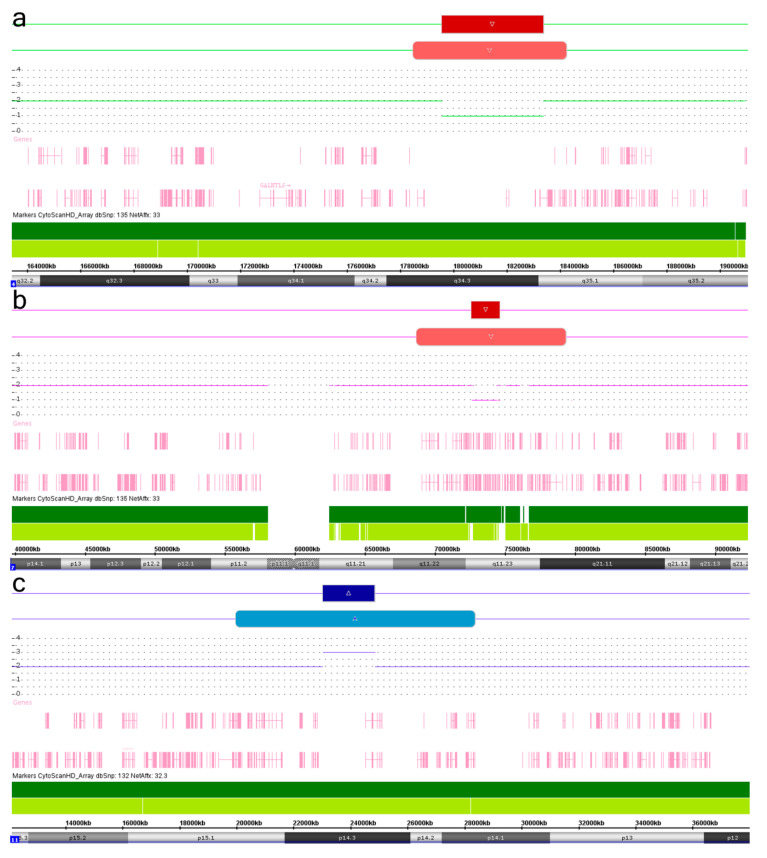
SNP-array analysis of colocalized regular/mosaic chromosome imbalances in an individual (chromosomal loci suggested to be affected by chromohelkosis or chromohelkosis imbalances): (**A**) regular/mosaic deletions at 4q34.3q35.1; (**B**) regular/mosaic deletions at 7q11.23/7q11.22q21.11; (**C**) regular/mosaic duplications at 11p14.3. Dark red—regular deletions; light red—mosaic deletions; dark blue—regular duplication; light blue—mosaic duplication.

Molecular karyotyping of the Russian cohort of children with neurodevelopmental disorders and congenital malformations [21,22], which currently includes 612 individuals, has been performed by high resolution SNP-array using Affymetrix CytoScan platform (HD). During the analysis, we observed concomitant regular and mosaic chromosome imbalances located at the same chromosomal locus or encompassed similar genomic regions in 47 individuals (7.7%). These colocalized structural chromosome abnormalities manifested as deletions or duplications with mosaic imbalances presenting mainly larger than regular ones. Accordingly, we have suggested that the colocalizations are not coincidental, representing, therefore, a snapshot of a possible dynamic process, which starts as a regular chromosomal imbalance (CNV) and proceeds with the formation of mosaic imbalances by a kind of “wreckage” at both breakpoints of the regular one. Hence, regular structural genomic variants are likely to be the first step initiating genomic instability (GIN) at adjacent chromosomal loci (genomic regions). The instability is likely to produce a larger rearrangement through defective DNA damage response and/or reparation (duplication—elongation by erroneous reparation; deletion—loss of chromosome parts). To define these colocalized chromosome abnormalities in an individual and to name the process producing SMC and CIN/GIN from regular genomic/chromosomal changes (CNV) by a single term, we have introduced the neologism “chromohelkosis”, which literally means “chromosome ulceration or ulcer” (from the Greek words “chromo” designating “chromosome” and “helkosis” derived from helkos (ἕλκος), which means “ulceration”, “ulcer”, or “open wound”). Consequently, the process is designated as chromohelkosis, whereas colocalized regular and mosaic chromosomal changes are designated as chromohelkosis imbalances. We intentionally composed the word “chromohelkosis” to mimic chromothripsis (chromosome shattering characterized by extensive rearrangements and an oscillating pattern of DNA copy numbers), chromoanasynthesis (local chromosome shattering associated with a random restitching of chromosomal fragments), and chromoanagenesis (complex rearrangements at one/several chromosomal loci produced by a catastrophic event) [4,5,6], inasmuch as it also seems to be a common mechanism for somatic chromosome rearrangements and CIN. Figure 1 shows three examples of chromosomal loci affected by chromohelkosis.

Chromohelkosis imbalances have been found to affect loci of almost all chromosomes apart from chromosomes 6, 8, 18–21, and Y. There have been detected 26 duplications and 21 deletions (55/45). Sizes of regular chromosome imbalances have been found to be highly variable from small CNV (tens to hundreds of kbp) to partial trisomies and monosomies (large chromosome deletions/duplications, e.g., >20 Mb). Mosaic chromosome imbalances have been generally more than 2 Mb, except one case (0.87 Mb). Details on the chromohelkosis imbalances are given in Table 1.

**Figure 2:**
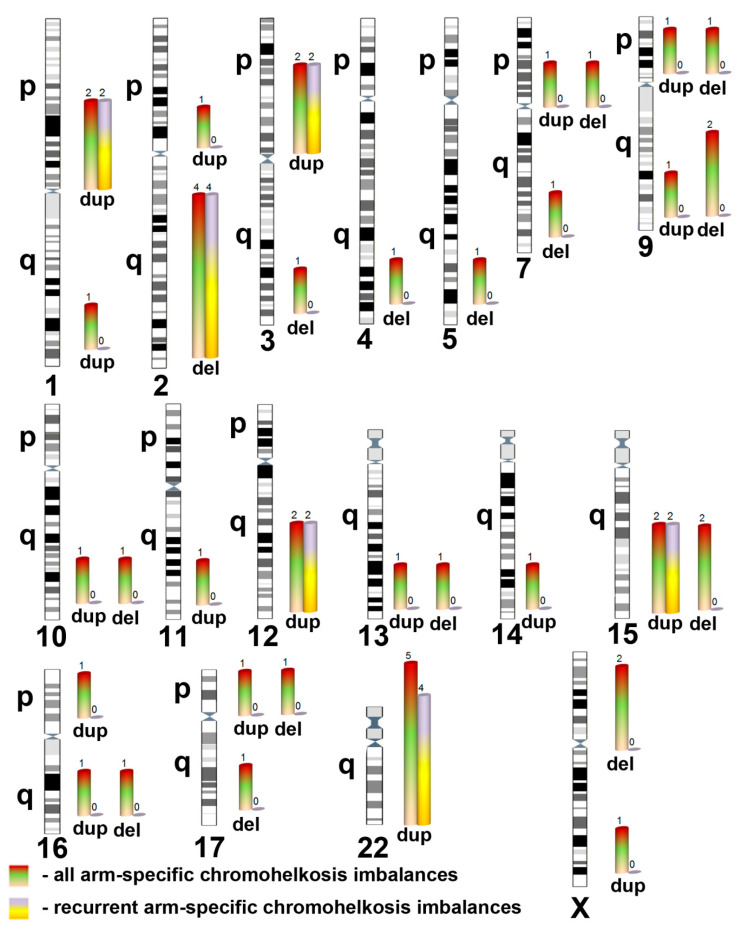
Spectrum of chromohelkosis imbalances classified according to chromosome arms and recurrence at the same chromosomal locus, encompassing the same genomic region (del—deletion; dup—duplication). Two differently colored bars show number of chromohelkosis imbalances per chromosome. The scale of bar length is the same for all chromosomes, whereas the scale of chromosomes is conserved.

Five chromohelkosis imbalances were recurrent, i.e., non-random variations at same genomic regions (Figure 2). This observation allowed us to conclude that specific genome architecture is likely to be involved in chromohelkosis as in germline and somatic variations mediating genomic disorders [8,13,14,15,16]. Additionally, reciprocal chromohelkosis imbalances (i.e., chromohelkosis deletions and duplications at the same chromosomal locus/genomic region) at 9p24.3, 9q34.13q34.3, and 15q11.2 have been revealed (Table 1). This observation additionally suggests that specificity of local genomic architecture makes the loci susceptible to genomic/chromosomal rearrangements) [13,14]. However, to provide further evidence and a molecular/cellular basis for chromohelkosis, additional analyses of molecular karyotyping data appeared to be required.

To characterize the spectrum of chromohelkosis imbalances, we used the ratio between sizes of regular and mosaic chromosome abnormalities. According to this parameter, cases were divided in quartiles (Q_1_—the smallest ratio/the longest distance between breakpoints of regular and mosaic imbalances; Q_4_—the largest ratio/the shortest distance between breakpoints of regular and mosaic imbalances). The first quartile (Q_1_) comprised 27 (57%) chromohelkosis imbalances (1p (*n* = 2), 1q, 2p, 2q (*n* = 4), 3q(del), 7q, reciprocal 9p(del), and 9p(dup), reciprocal 9q(del) and 9q(dup), 11p, 12q (*n* = 2), 13q, 15q(dup) (*n* = 2), 16q(del), 16q(del), 17p(dup), 17q, Xp, Xq), whereas the remaining quartiles (Q_2_–Q_4_) comprised 20 chromohelkosis imbalances (3p (*n* = 2), 4q, 5q, reciprocal 7p(del), and 7p(dup), 9q(del), 10q (*n* = 2), 14q, 15q(del) (*n* = 2), 16p, 22q (*n* = 5), Xp). The distribution of chromohelkosis imbalances demonstrates a large segregation towards abnormalities with dramatically increased sizes of mosaic ones. Q_1_-imbalances are, therefore, more likely to be associated with a higher rate of local GIN both at the breakpoints and at the affected chromosomal loci. Correlations between the ratio and patient age, chromosomal localization, or imbalance specificity (deletion/duplication) have not been found. Thus, individual susceptibility to chromohelkosis has been proposed as the mechanism of the occurrence and distribution in favor of Q_1_. As described previously, such a kind of susceptibility may be a result of alterations to molecular and cellular pathways safeguarding genome stability [12,23,24]. Recently, a model for pathway-based classification of genomic burden (CNV burden) resulting in CIN/GIN and SCM was proposed [25]. Using this model and a novel bioinformatic method for pathway-based CNV/gene prioritization [26], we evaluated CNV burden effect on pathways required for genome stability maintenance (e.g., DNA reparation and damage response, programmed cell death, cell cycle regulation, cancer-related pathways, etc.). Accordingly, we retrieved ontologies of genes affected by regular CNV and chromosome imbalances in the genome stability context and identified I_pp_ (index of pathway prioritization): I_pp_(Q_1_) = 2.4 and 46 pathways; I_pp_(Q_2_–Q_4_) = 1.7 and 27 pathways (for more details, see [26]). We observed a statistically significant difference between Q_1_ and Q_2_–Q_4_ in enrichment of genome stability maintenance pathways (*Z*-test, *p* < 0.001). As a result, we concluded that non-random distribution of chromohelkosis imbalances is likely to be caused by individual susceptibility to GIN/CIN produced by alterations to pathways safeguarding genome stability. In other words, a kind of saturation in CNV encompassing genes involved in these pathways may result in chromohelkosis. Moreover, a correlation between a higher CNV burden and a measure of chromohelkosis progression appears to exist. It is highly likely that the measure of chromohelkosis progression may be connected to repeat DNA, which is involved in genome organization and stability [27] (discussed hereafter).

The appreciable difference between the regular and mosaic (colocalized) rearrangements is reflected in gene content of a chromohelkosis imbalance (Supplementary Table S1). For instance, three individuals exhibited chromohelkosis imbalances characterized by the lack of functionally annotated genes in regions affected by regular rearrangements (chromohelkosis imbalance at 11p and twice at 2q). In addition, numerous cases demonstrated phenotypic outcomes associated with mosaic rearrangements but not with regular ones (e.g., rearrangements at 1p, 9q, 15q, 16q, 17p, and 22q). However, it is noteworthy that these phenotypic manifestations are milder than those observed in recognizable chromosomal (microdeletion or microduplication) syndromes. These observations might be used to explain extensive variability of SCM phenotypes, as suggested previously [8,28,29,30]. Here, it is recognized that chromohelkosis may have a diagnostic relevance comparable to chromothripsis, chromoanasynthesis, and chromoanagenesis.

**Figure 3:**
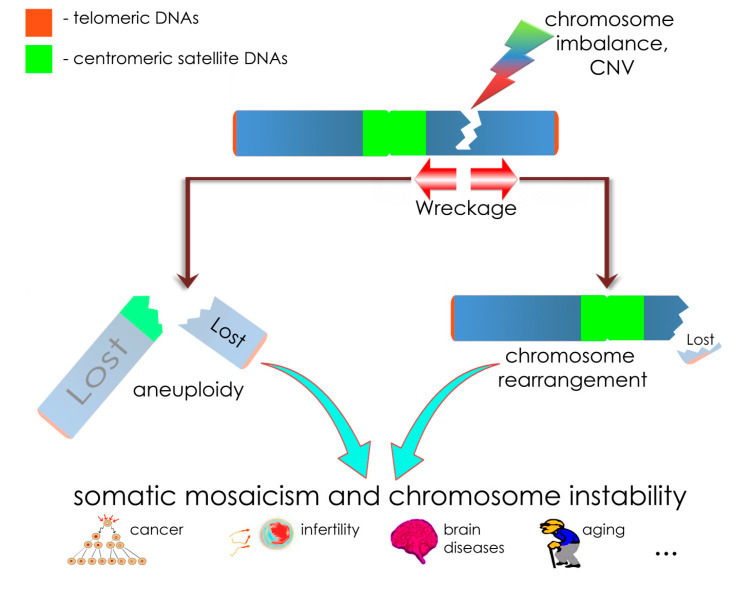
Schematic depiction of chromohelkosis due to a deletion and suggested outcomes relevant to the cytogenomic “theory of everything” (discussed hereafter). Chromosomal imbalances or CNV manifesting as deletions are able to cause instability at the sequence level (GIN) at the breakpoints. Consequently, wreckage may occur through altered DNA damage response and reparation, which cause progressive loss of chromosomal DNA localized at the breakpoints. According to the theory, (centromeric) satellite DNA protects chromosomes from propagation of chromohelkosis in the same way as telomeres do. However, the protection may fail to initiate two scenarios: (i) the distal chromosome part is wrecked and lost, when telomeres are affected; as a result, somatic chromosome rearrangements are formed (on the right-hand side); (ii) the proximal chromosome part is wrecked and, if centromeric satellite DNA fails to protect the centromere, the whole chromosome is lost (i.e., aneuploidy/monosomy) (on the left-hand side). This cascade of CIN/GIN processes results in somatic mosaicism and CIN, which are mechanisms for cancer, infertility, brain diseases, aging, and, probably, other morbid conditions. To depict a biological basis of chromohelkosis, we have used parts of figures from our previous articles distributed under the terms of the Creative Commons Attribution License [28,39].

Among others, chromohelkosis may represent a model of somatic genome variability (i.e., SMC, GIN, and CIN). We have suggested that chromohelkosis deletions produce local instability at the breakpoints leading to progressive loss of adjacent chromosomal regions. As a result, mosaic deletions are larger than regular ones. This mechanism is similar to some extent to those proposed for explaining pathways from chromosome fragile sites to somatic chromosomal aberrations and CIN to some extent [31,32]. Moreover, fragile sites affect replication timing producing DNA flexibility peaks and stress inducible asynchrony at the breakage to produce GIN or CIN [31]. This process mimics “wreckages” at the breakpoints of chromohelkosis imbalances. However, chromohelkosis is able to lead to the loss of a larger chromosomal region due to the dynamic nature. Chromohelkosis affecting the centromere would result in a loss of the whole chromosome (mosaic aneuploidy/monosomy). Alterations to centromere stability (scission) have been recently shown to be a mechanism for GIN/CIN [33]. It is also known that CIN and GIN may be mediated by pathways involving centromeric DNA (e.g., classical/alpha satellite DNA) [3,20,33,34]. Although functional significance of centromeric satellite DNA (constitutive heterochromatin) remains a matter of further investigation, numerous studies indicate the involvement in granting genome/chromosome stability [35,36,37]. Satellite DNA is suggested either to mediate GIN or to protect from alterations to chromosome structure [27]. Here, we propose a role of centromeric satellite DNA similar to telomeric DNA [38], which seems to play a role in CIN caused by chromohelkosis, as well. Thus, centromeric satellite DNA protects centromere from chromohelkosis “wreckage”. Similarly, telomeric DNA is proposed to play the canonical role of protecting chromosomal ends from chromohelkosis “wreckage”. The failure of centromeric satellite DNA to protect centromere from the “wreckage” would result in chromosomal loss (aneuploidy). The failure of telomeric DNA to protect chromosomal ends from “wreckage” would result in chromosomal rearrangements. Figure 3 schematically shows suggested mechanisms of chromohelkosis mediated by deletions.

The elongation of duplicated regions might be mediated by fails of chromosomal DNA reparation at the breakpoints progressively occurring during each fail. Related mechanisms for CIN have been already described in cancers [20,23]. These duplications may also involve repeat (satellite) DNA as a driving force for chromohelkosis progression [27]. Probably, the consequences of chromohelkosis mediated by deletions may be used to explain a slight preponderance of duplications over deletions. More precisely, mosaic deletion is more likely to disappear, becoming aneuploidy (chromosome loss), whereas mosaic duplication is likely to become a larger one.

SMC and CIN are important contributors to development, homeostasis, interindividual diversity, and disease. To be more exact, SMC and CIN are integrated parts of human prenatal development [40,41,42], aging [43,44], cancer [24,45,46], interindividual/intercellular genome diversification, and disease [40,47,48,49,50,51,52]. SMC and CIN are considered a major focus of basic and diagnostic research for providing therapeutic opportunities in disease and aging [29,52,53,54]. Therefore, it is hard to overestimate the role of SMC and CIN. Our current observation hallmarks a common mechanism of somatic genome variability produced by chromosomal imbalances and CNV. Taking into account a kind of omnipresence of chromosomal variations and CNV [7,28,47,53], the chromohelkosis-based pathway to SMC/CIN might underlie a cytogenomic theory, which would be relevant to numerous areas of genetics, genomics, and chromosome research. In our opinion, such a theory resembles the elusive theory of everything described by Stephen Hawking and Leonard Mlodinow [55], because both create a temptation to explain almost everything in a given research area. To decrease the temptation, we used metaphoric (ironic) designation “the cytogenomic theory of everything” for the system explaining chromohelkosis contribution to SCM/CIN. Finally, recognizing empirical and theoretical weaknesses of our contribution, we nevertheless insist that chromohelkosis is a process to explore for understanding structural variability and flexibility of the genome.

## 3. Materials and Methods

### 3.1. Patients and Samples

Molecular karyotyping was performed to identify chromosomal abnormalities and CNV in the Russian cohort of children with neurodevelopmental disorders (intellectual disability, autism, epilepsy) and congenital anomalies (612 individuals), which has been clinically described in previous studies [21,22]. This study was approved by the Ethics Committee of the Veltischev Research and Clinical Institute for Pediatrics of the Pirogov Russian National Research Medical University, Ministry of Health of Russian Federation, Moscow, Russia (AAAA—A18—118051590122—7 # 6, 19 June 2019). Written informed consent was obtained from the patients’ parents.

### 3.2. SNP-Array

Molecular karyotyping was performed by CytoScan HD Arrays (Affymetrix, Santa Clara, CA, USA) consisting of about 2.7 million markers. All the procedures have been repeatedly described in detail previously [21,22,56,57]. Cytogenomic variations were visualized using the Affymetrix ChAS (Chromosome Analysis Suite) software (CytoScan^®^ HD Array Version 4.1.0.90/r29400). The reference sequence was GRCh37/hg19.

### 3.3. Bioinformatic Analysis

An original approach to prioritization of CNV, candidate genes, and processes using molecular karyotyping data was carried out as described earlier [58]. The procedure is an ontology-based gene filtering/ranking with fusion of data acquired from databases dedicated to clinical genetics, genomics, epigenetics (gene expression), proteomics (interactome), and metabolome. Additionally, genomic data were analyzed by obtaining the ratio between the size of regular and mosaic imbalances. Using this ratio, imbalances were divided in quartiles. The data have been also used for pathway analysis.

#### Pathway Analysis

Pathway analysis was performed using CNVariome concept and data laundering protocol, which were recently described in detail [25,26]. The enrichment by genome stability maintenance pathways was additionally analyzed using statistical *Z*-test.

## 4. Conclusions

In summary, we observed co-occurrence of regular and mosaic imbalances (deletions/duplications) at same chromosomal loci during molecular karyotyping. Data analysis allowed us to propose a mechanism for SMC and CIN — chromohelkosis (chromosome ulceration/open wound). Further exploration of this mechanism has led to suggesting a function for classical/alpha satellite DNA (protection from the wreckage towards the centromere) and cytogenomic “theory of everything”, a metaphorically termed system explaining chromohelkosis contribution to SCM and CIN. Chromohelkosis is proposed to be the result of a cumulative effect of chromosomal imbalance/CNV (or, probably, other alterations to chromosomal structure) and mutational/CNV burden, which alters pathways of genome stability maintenance. These combinations of genomic variations would be critical for chromohelkosis dynamics and the levels of SCM and CIN. Thus, forthcoming studies are to uncover the spectrum of genomic variations leading to chromohelkosis to show whether this process is restricted to specific chromosomal imbalances or CNV.

## Figures and Tables

**Table 1:** Colocalized regular/mosaic chromosome imbalances and copy number variations (CNV) marking chromosomal loci affected by chromohelkosis (chromohelkosis imbalances).

Chromosomal Localization		Deletion (del)/Duplication (dup)	Genomic Localization ^1^		Size (Mb), Regular	Size (Mb), Mosaic	Mosaicism Rate (%) ^2^
Regular	Mosaic		Regular	Mosaic			
1p36.32	1p36.33p36.32	Dup	2,793,846–3,123,524	849,466–3,586,707	0.329	2.737	20
1p36.33	1p36.33p36.32	Dup	1,134,091–1,207,463	849,466–5,278,786	0.073	4.429	25
1q21.1q21.2	1p12q21.2	Dup	146,003,044–147,398,560	118,506,747–149,965,913	1.395	31.459	20
2p25.3	2p25.3	Dup	1,611,691–1,861,548	12,770–3,007,511	0.249	2.994	25
2q22.2 q22.3	2q22.1q23.3	Del	143,410,303–145,299,945	140,410,739–150,635,360	1.889	10.224	50
2q23.1q23.3	2q22.2q24.1	Del	148,851,963–151,316,465	143,753,727–155,408,790	2.464	11.655	40
2q23.1q23.3	2q22.2q24.1	Del	149,073,384–151,886,100	144,007,224–156,393,001	2.812	12.385	45
2q24.1	2q23.3q24.2	Del	155,684,576–157,919,431	151,497,654–162,200,234	2.234	10.702	35
3p26.1	3p26.3p26.1	Dup	4,311,166–7,256,278	2,788,170–8,587,443	2.945	5.799	40
3p26.3p26.2	3p26.3p26.1	Dup	1,839,722–3,372,758	61,891–4,693,249	1.533	4.631	60
3q26.2q26.31	3q26.1q26.31	Del	170,316,791–171,650,195	165,957,466–175,300,706	1.333	9.343	30
4q34.3q35.1	4q34.3q35.1	Del	179,568,373–183,377,810	178,503,425–184,251,370	3.809	5.747	55
5q35.2q35.3	5q35.1q35.3	Del	175,029,372–177,324,736	171,538,904–180,719,789	3.395	9.180	30
7p22.1p15.2	7p22.1p15.2	Dup	4,783,314–26,275,210	4,790,968–26,522,153	21.491	21.731	80
7p22.2p21.3	7p22.3p21.3	Del	3,235,409–7,970,015	43,360–8,320,635	4.734	8.277	20
7q11.23	7q11.22q21.11	Del	72,612,042–74,610,673	68,665,592–79,305,748	1.998	10.640	40
9p24.3	9p24.3p24.2	Dup	203,861–823,845	203,861–2,593,900	0.619	2.390	30
9p24.3	9p24.3	Del	203,861–410,357	203,861–1,074,830	0.206	0.870	25
9q22.31q22.33	9q22.31q22.33	Del	96,109,697–99,973,789	95,891,880–100,145,863	3.864	4.253	70
9q34.3	9q34.13q34.3	Dup	139,053,501–139,435,356	134,317,328–141,020,389	0.381	6.703	30
9q34.3	9q34.13q34.3	Del	139,784,913–141,020,389	135,282,452–141,020,389	1.235	5.737	40
10q21.1	10q11.23q21.1	Dup	53,156,807–57,931,080	52,693,425–58,936,553	4.774	6.243	75
10q23.1q23.2	10q23.1q23.2	Del	86,412,180–88,502,670	85,638,142–89,465,109	2.090	3.826	50
11p14.3	11p14.3	Dup	23,032,300–24,850,872	19,983,179–28,380,051	1.819	8.396	25
12q24.33	12q24.33	Dup	129,804,153–130,492,863	129,577,575–133,777,902	0.688	4.200	20
12q24.33	12q24.33	Dup	129,803,493–130,485,474	130,035,491–133,777,902	0.681	3.742	20
13q12.11	13q11q12.11	Dup	21,683,950–22,155,929	19,436,287–22,422,460	0.471	2.986	20
13q34	13q34	Del	114,085,478–115,107,733	110,963,086–115,107,733	1.022	4.144	40
14q32.2	14q32.13q32.2	Dup	99,153,952–101,024,454	95,563,168–100,095,249	1.870	4.532	20
15q11.2	15q11.2	Dup	22,770,421–23,082,328	22,770,421–25,083,880	0.311	2.313	20
15q11.2	15q11.2	Dup	22,770,421–23,288,350	22,770,421–25,318,376	0.517	2.547	25
15q11.2q13.1	15q11.2q13.1	Del	22,770,421–29,021,034	22,770,421–28,373,187	5.732	5.602	85
15q13.2q13.3	15q13.1q14	Del	30,913,573–32,914,239	28,394,840–36,544,674	2.518	8.149	25
16p11.2q11.2	16p11.2q12.1	Dup	32,038,693–46,463,769	34,448,198–51,124,520	14.425	16.676	20
16q23.1	16q22.3q23.3	Dup	77,496,014–78,916,839	73,357,720–82,335,001	1.420	8.977	30
16q24.3	16q24.2q24.3	Del	89,683,742–90,155,062	87,157,300–90,155,062	0.471	2.997	25
17p12	17p13.1p11.2	Dup	14,082,944–15,479,940	10,219,298–17,108,606	1.396	6.889	20
17p13.3	17p13.3p13.2	Del	525–1,323,904	525–4,375,742	1.323	4.375	40
17q25.3	17q25.3	Del	80,396,463–81,041,938	77,947,778–81,041,938	0.645	3.094	30
22q11.21	22q11.1q11.22	Dup	18,974,541–21,800,797	17,398,811–23,374,206	2.826	5.975	45
22q11.21	22q11.1q11.21	Dup	18,649,189–20,311,810	16,888,899–22,034,665	1.662	5.145	40
22q11.21	22q11.1q11.22	Dup	18,979,345–21,465,659	16,888,899–23,410,418	2.486	6.521	50
22q11.21	22q11.1q11.23	Dup	18,916,842–21,465,659	16,888,899–23,535,339	2.548	6.646	50
22q13.2q13.31	22q13.2q13.31	Dup	43,337,317–46,575,998	42,018,242–44,860,024	3.238	2.841	50
Xp22.31	Xp22.32p22.2	Del	6,784,550–7,495,395	4,931,788–9,634,138	0.710	4.702	25
Xp21.1	Xp21.1p11.4	Del	32,881,263–35,187,430	31,875,672–38,716,579	2.306	6.840	50
Xq28	Xq28	Dup	153,747,685–153,761,134	147,843,549–152,036,631	0.013	4.193	20

^1^ GRCh37/hg19. ^2^ mosaicism rates according to Chromosome Analysis Suite (ChAS) software (Affymetrix).

## Supplementary Materials

The following are available online at https://www.mdpi.com/1422-0067/21/21/8328/s1.

## Abbreviations

ChAS	Chromosome Analysis Suite
CIN	Chromosomal instability
CNV	Copy number variations
Del	deletion (tables & figures)
dup	duplication (tables & figures)
GIN	Genome instability
I_PP_	Index of pathway prioritization
SCM	Somatic chromosomal mosaicism
SNP	Single nucleotide polymorphism
