# Supplementary material for: The Cytogenomic “Theory of Everything”: Chromohelkosis May Underlie Chromosomal Instability and Mosaicism in Disease and Aging
Source: Int J Mol Sci. 2020 Nov 6;21(21):8328. doi: 10.3390/ijms21218328 (PMC7664247; doi:10.3390/ijms21218328)
Supplement: Supplementary file 1 [file ijms-21-08328-s001.pdf]

International Journal of *Molecular Sciences*

MDPI

Article

The Cytogenomic "Theory of Everything": Chromohelkosis May Underlie Chromosomal Instability and Mosaicism in Disease and Aging

Ivan Y. Iourov [1,2,3,*], Svetlana G. Vorsanova [1,2], Yuri B. Yurov [1,2], Maria A. Zelenova [1,2], Oxana S. Kurinnaia [1,2], Kirill S. Vasin [1,2] and Sergei I. Kutsev [4]

1 Mental Health Research Center, Moscow 117152, Russia; ivan.iourov@gmail.com (I.Y.I.); svorsanova@mail.ru (S.G.V.); y_yurov@yahoo.com (Y.B.Y.); maria_zelenova@yahoo.com (M.A.Z.); kurinnaiaos@mail.ru (O.S.K.); vasin-ks@rambler.ru (K.S.V.)
2 Veltischev Research and Clinical Institute for Pediatrics of the Pirogov Russian National Research Medical University, Ministry of Health of Russian Federation, Moscow 125412, Russia;
3 Department of Medical Biological Disciplines, Belgorod State University, Belgorod 308015, Russia;
4 Research Centre for Medical Genetics, Moscow 115522, Russia; kutsev@mail.ru
* Correspondence: ivan.iourov@gmail.com

Received: 22 October 2020; Accepted: 4 November 2020; Published: date

Supplementary Table 1. Disease-associated genes located between breakpoints of mosaic and regular chromosome imbalances (CNV).

Chromosomal Localization (Mosaic)	Genes Located between Proximal Breakpoints	Genes Located between Distal Breakpoints
1p36.33p36.32	—	*ISG15* [OMIM:147571] (immunodeficiency 38 [OMIM:616126]), *AGRN* [OMIM:103320] (myasthenic syndrome, congenital, 8, with pre- and postsynaptic defects [OMIM:615120]), *TNFRSF4* [OMIM:600315] (immunodeficiency 16 [OMIM:615593]), *B3GALT6* [OMIM:615291] (Ehlers-Danlos syndrome, progeroid type, 2 [OMIM:615349], spondyloepimetaphyseal dysplasia with joint laxity, type 1, with or without fractures [OMIM:271640]), *DVL1* [OMIM:601365] (Robinow syndrome, autosomal dominant 2 [OMIM:616331]), *TMEM240* [OMIM:616101] (spinocerebellar ataxia 21 [OMIM:607454]), *GABRD* [OMIM:137163] (epilepsy, generalized, with febrile seizures plus, type 5, susceptibility to [OMIM:613060],

		epilepsy, idiopathic generalized, 10 [OMIM:613060], epilepsy, juvenile myoclonic, susceptibility to [OMIM:613060]), *SKI* [OMIM:164780] (Shprintzen-Goldberg syndrome [OMIM:182212]), *PEX10* [OMIM:602859] (peroxisome biogenesis disorder 6A (Zellweger) [OMIM:614870], Peroxisome biogenesis disorder 6B [OMIM:614871])
1p36.33p36.32	*TAS1R3* [OMIM:605865], *DVL1* [OMIM:601365] (Robinow syndrome, autosomal dominant 2 [OMIM:616331]), *TMEM240* [OMIM:616101] (spinocerebellar ataxia 21 [OMIM:607454]), *GABRD* [OMIM:137163] (epilepsy, generalized, with febrile seizures plus, type 5, susceptibility to [OMIM:613060], epilepsy, idiopathic generalized, 10 [OMIM:613060], epilepsy, juvenile myoclonic, susceptibility to [OMIM:613060]), *SKI* [OMIM:164780] (Shprintzen-Goldberg syndrome [OMIM:182212]), *PEX10* [OMIM:602859] (peroxisome biogenesis disorder 6A (Zellweger) [OMIM:614870], peroxisome biogenesis disorder 6B [OMIM:614871]), *PRDM16* [OMIM:605557] (cardiomyopathy, dilated, *1LL* [OMIM:615373], left ventricular noncompaction 8 [OMIM:615373]), *TP73* [OMIM:601990] (neuroblastoma), *CEP104* [OMIM:616690] (Joubert syndrome 25 [OMIM:616781])	*ISG15* [OMIM:147571] (immunodeficiency 38 [OMIM:616126]), *AGRN* [OMIM:103320] (myasthenic syndrome, congenital, 8, with pre- and postsynaptic defects [OMIM:615120])
2p25.3	*MYT1L* [OMIM:613084](mental retardation, autosomal dominant 39 [OMIM:616521])	*TPO* [OMIM:606765] (thyroid dyshormonogenesis 2A [OMIM:274500])
2q22.2q24.1	*ZEB2* [OMIM:605802] (Mowat-Wilson syndrome [OMIM: 235730]), *ORC4* [OMIM:603056] (Meier-Gorlin syndrome 2 [OMIM:613800]), *MBD5* [OMIM:611472] (mental retardation, autosomal dominant 1 [OMIM:156200])	*NEB* [OMIM:161650](nemaline myopathy 2, autosomal recessive), *CACNB4* [OMIM:601949] (episodic ataxia, type 5 [OMIM:613855], epilepsy, idiopathic generalized, susceptibility to, 9 [OMIM:607682], epilepsy, juvenile myoclonic, susceptibility to, 6 [OMIM:607682])
2q22.2q24.1	*KYNU* [OMIM:605197] (hydroxykynureninuria [OMIM:236800]), *ZEB2* [OMIM:605802] (Mowat-Wilson	*NEB* [OMIM:161650] (nemaline myopathy 2, autosomal recessive [OMIM:256030]),

	syndrome [OMIM:235730]), *ORC4* [OMIM:603056] (Meier-Gorlin syndrome 2 [OMIM:613800]), *MBD5* [OMIM:611472] (Mental retardation, autosomal dominant 1 [OMIM:156200])	*CACNB4* [OMIM:601949] (episodic ataxia, type 5 [OMIM:613855], epilepsy, idiopathic generalized, susceptibility to, 9 [OMIM:607682], epilepsy, juvenile myoclonic, susceptibility to, 6 [OMIM:607682])
3p26.3p26.1	*SUMF1* [OMIM:607939] (multiple sulfatase deficiency [OMIM:272200]), *ITPR1* [OMIM:147265] (gillespie syndrome [OMIM:206700], spinocerebellar ataxia 15 [OMIM:606658], spinocerebellar ataxia 29, congenital nonprogressive [OMIM:117360])	—
3p26.3p26.1	—	*CRBN* [OMIM:609262] (mental retardation, autosomal recessive 2 [OMIM:607417])
3q26.1q26.31	*PDCD10* [OMIM:609118] (cerebral cavernous malformations 3 [OMIM:603285]), *SERPINI1* [OMIM:602445] (encephalopathy, familial, with neuroserpin inclusion bodies [OMIM:604218]), *MECOM* [OMIM:165215] (radioulnar synostosis with amegakaryocytic thrombocytopenia 2 [OMIM:616738]), *TERC* [OMIM:602322] (dyskeratosis congenita, autosomal dominant 1[OMIM: 127550], aplastic anemia [OMIM:614743], pulmonary fibrosis, idiopathic, susceptibility to} [OMIM:614743], *SLC7A14* [OMIM:615720](retinitis pigmentosa 68 [OMIM:615725])	*GHSR* [OMIM:601898] (growth hormone deficiency, isolated partial [OMIM:615925]), *SPATA16* [OMIM:609856] (spermatogenic failure 6 [OMIM:102530])
4q34.3q35.1	—	*TENM3* [OMIM:610083] (microphthalmia, isolated, with coloboma 9 [OMIM:615145])
5q35.1q35.3	*SH3PXD2B* [OMIM:613293] (Frank-ter Haar syndrome [OMIM: 249420]), *NKX2-5* [OMIM:600584] (atrial septal defect 7, with or without AV conduction defects [OMIM:108900], conotruncal heart malformations, variable [OMIM:217095], hypoplastic left heart syndrome 2 [OMIM:614435], hypothyroidism, congenital nongoitrous, 5 [OMIM:225250], tetrology of Fallot [OMIM:187500], ventricular septal defect 3 [OMIM:614432]), *MSX2* [OMIM:123101] (craniosynostosis, type 2 [OMIM:604757], parietal foramina 1 [OMIM:168500], parietal foramina with cleidocranial dysplasia [OMIM:168550]), *DRD1* [OMIM:126449]	*PROP1* [OMIM:601538] (pituitary hormone deficiency, combined, 2), *NOLA2* [OMIM:606470] (dyskeratosis congenita, autosomal recessive 2), *HNRNPAB* [OMIM:602688], *AGXT2L2* [OMIM:614683] (phosphohydroxylysinuria [OMIM:615011]), *GRM6* [OMIM:604096] (night blindness, congenital stationary (complete), 1B, autosomal recessive [OMIM:257270]), *ADAMTS2* [OMIM:604539] (Ehlers-Danlos syndrome, type VIIC [OMIM: 225410]), *LTC4S* [OMIM:246530] (leukotriene C4 synthase

		deficiency [OMIM:614037]), *SQSTM1* [OMIM:601530] (Paget disease of bone [OMIM:602080]), *FLT4* [OMIM:136352] (hemangioma, capillary infantile, somatic [OMIM:602089], lymphedema, hereditary, IA [OMIM:153100])
7p22.1p15.2	*SNX10* [OMIM:614780] (osteopetrosis, autosomal recessive 8 [OMIM:615085])	—
7p22.2p21.3	*GLCCI1* [OMIM:614283] ({glucocorticoid therapy, response to} [OMIM:614400]),	*FAM20C* [OMIM:611061] (Raine syndrome [OMIM: 259775]), *HEATR2* [OMIM:614864](ciliary dyskinesia, primary, 18 [OMIM:614874]), *MAD1L1* [OMIM:602686](lymphoma, somatic, prostate cancer, somatic [OMIM:176807]), *BRAT1* [OMIM:614506] (rigidity and multifocal seizure syndrome, lethal neonatal [OMIM:614498]), *CARD11* [OMIM:607210] (B-cell expansion with NFKB and T-cell anergy [OMIM:616452], immunodeficiency 11A [OMIM:615206], immunodeficiency 11B with atopic dermatitis [OMIM:617638])
7q11.22q21.11	*AUTS2* [OMIM:607270](mental retardation, autosomal dominant 26 [OMIM:615834])	*POR* [OMIM:124015] (Antley-Bixler syndrome with genital anomalies and disordered steroidogenesis [OMIM:201750], disordered steroidogenesis due to cytochrome P450 oxidoreductase [OMIM:613571]), *MDH2* [OMIM:154100] (epileptic encephalopathy, early infantile, 51 [OMIM:617339]), *HSPB1* [OMIM:602195] (Charcot-Marie-Tooth disease, axonal, type 2F [OMIM:606595], neuropathy, distal hereditary motor, type IIB [OMIM:608634]), *YWHAG* [OMIM:605356] (epileptic encephalopathy, early infantile, 56 [OMIM:617665]), *SRCRB4D* [OMIM:607639], *ZP3* [OMIM:182889] (oocyte maturation defect 3 [OMIM: 17712]), *PTPN12* [OMIM:600079] (colon cancer, somatic

		[OMIM:114500]), *MAGI2* [OMIM:606382] (nephrotic syndrome 15 [OMIM:617609])
9p24.3p24.2	*SMARCA2* [OMIM:600014] (Nicolaides-Baraitser syndrome [OMIM:601358]	—
9q34.13q34.3	*POMT1* [OMIM:607423] (muscular dystrophy-dystroglycanopathy (congenital with brain and eye anomalies), type A, 1[OMIM:236670], muscular dystrophy-dystroglycanopathy (congenital with mental retardation), type B, 1 [OMIM:613155], muscular dystrophy-dystroglycanopathy (limb-girdle), type C, 1 [OMIM:609308]), *SETX* [OMIM:608465] (amyotrophic lateral sclerosis 4, juvenile [OMIM:602433], spinocerebellar ataxia, autosomal recessive 1 [OMIM:606002]), *TSC1* [OMIM:605284] (focal cortical dysplasia, Taylor balloon cell type [OMIM:607341], lymphangioleiomyomatosis [OMIM:606690], tuberous sclerosis-1 [OMIM:191100]), *GFI1B* [OMIM:604383] (bleeding disorder, platelet-type, 17 [OMIM:187900]), *CEL* [OMIM:114840] (maturity-onset diabetes of the young, type VIII [OMIM:609812]), *SURF1* [OMIM:185620] (Leigh syndrome, due to COX deficiency [OMIM:256000]), *ADAMTS13* [OMIM:604134] (thrombotic thrombocytopenic purpura, familial [OMIM:274150]), *ADAMTSL2* [OMIM:612277] (geleophysic dysplasia 1 [OMIM:231050]), *DBH* [OMIM:609312] (dopamine beta-hydroxylase deficiency [OMIM:223360], dopamine-beta-hydroxylase activity levels, plasma), *SARDH* [OMIM:604455] (sarcosinemia [OMIM:268900]), *COL5A1* [OMIM:120215] (Ehlers-Danlos syndrome, classic type [OMIM:130000]), *KCNT1* [OMIM:608167] (epilepsy, nocturnal frontal lobe, 5 [OMIM:615005], epileptic encephalopathy, early infantile, 14 [OMIM:614959])	*NOTCH1* [OMIM:190198] (Adams-Oliver syndrome 5 [OMIM:616028], aortic valve disease 1 [OMIM:109730]), *AGPAT2* [OMIM:603100] (lipodystrophy, congenital generalized, type 1 [OMIM:608594])
9q34.13q34.3	*TSC1* [OMIM:605284] (focal cortical dysplasia, type II, somatic [OMIM:607341], lymphangioleiomyomatosis [OMIM:606690], tuberous sclerosis-1 [OMIM:191100]), *CEL* [OMIM:114840] (maturity-onset diabetes of the young, type VIII [OMIM:609812]), *SURF1* [OMIM:185620] (Charcot-Marie-Tooth disease, type 4K [OMIM:616684], Leigh	

	syndrome, due to COX IV deficiency [OMIM:256000]), *ADAMTS13* [OMIM:604134] (thrombotic thrombocytopenic purpura, familial [OMIM:274150]), *ADAMTSL2* [OMIM:612277] (geleophysic dysplasia 1 [OMIM:231050]), *DBH* [OMIM:609312] (orthostatic hypotension 1, due to DBH deficiency [OMIM:223360]), *SARDH* [OMIM:604455] ([sarcosinemia] [OMIM:268900]), *COL5A1* [OMIM:120215] (Ehlers-Danlos syndrome, classic type, 1 [OMIM:130000]), *MRPS2* [OMIM:611971] (combined oxidative phosphorylation deficiency 36) [OMIM:617950], *SOHLH1* [OMIM:610224] (ovarian dysgenesis 5 [OMIM:617690], spermatogenic failure 32 [OMIM:618115]), *KCNT1* [OMIM:608167] (epilepsy, nocturnal frontal lobe, 5 [OMIM:615005], epileptic encephalopathy, early infantile, 14 [OMIM:614959]), *LHX3* [OMIM:600577] (pituitary hormone deficiency, combined, 3 [OMIM:221750]), *CARD9* [OMIM:607212] (candidiasis, familial, 2, autosomal recessive [OMIM:212050]), *PMPCA* [OMIM:613036] (spinocerebellar ataxia, autosomal recessive 2 [OMIM:213200]), *INPP5E* [OMIM:613037] (Joubert syndrome 1 [OMIM:213300], mental retardation, truncal obesity, retinal dystrophy, and micropenis [OMIM:610156]), *NOTCH1* [OMIM:190198] (Adams-Oliver syndrome 5 [OMIM:616028], aortic valve disease 1 [OMIM:109730]), *AGPAT2* [OMIM:603100] (lipodystrophy, congenital generalized, type 1 [OMIM:608594])	
12q24.33	—	*PUS1* [OMIM:608109] (mitochondrial myopathy and sideroblastic anemia 1), *P2RX2* [OMIM:600844] (deafness, autosomal dominant 41 608224), *POLE* [OMIM:174762] (FILS syndrome [OMIM:615139],], IMAGE-I syndrome [OMIM:618336], colorectal cancer, susceptibility to, 12 [OMIM:615083])
13q11q12.11	*GJA3* [OMIM:121015] (cataract 14, multiple types [OMIM:601885]), *GJB2* [OMIM:121011] (Bart-Pumphrey syndrome [OMIM:149200], 3 Deafness, autosomal dominant 3A [OMIM:601544], Deafness, autosomal	*FGF9* [OMIM:600921] (multiple synostoses syndrome 3 [OMIM:612961])

13q34	recessive 1A [OMIM:220290], Hystrix-like ichthyosis with deafness [OMIM:602540], Keratitis-ichthyosis-deafness syndrome [OMIM:148210], Keratoderma, palmoplantar, with deafness [OMIM:148350], Vohwinkel syndrome 124500]), *GJB6* [OMIM:604418] (deafness, autosomal dominant 3B [OMIM:612643], deafness, autosomal recessive 1B [OMIM:612645], deafness, digenic GJB2/GJB6 [OMIM:220290], ectodermal dysplasia 2, Clouston type [OMIM:129500]) *COL4A2* [OMIM:120090](porencephaly 2 [OMIM:614483], hemorrhage, intracerebral, susceptibility to [OMIM:614519]), *CARS2* [OMIM:612800](combined oxidative phosphorylation deficiency 27 [OMIM:616672]), *ING1* [OMIM:601566](squamous cell carcinoma, head and neck, somatic [OMIM:275355]), *F7* [OMIM:613878](factor VII deficiency [OMIM:227500], myocardial infarction, decreased susceptibility to [OMIM:608446]), *F10* [OMIM:613872](factor X deficiency [OMIM:227600]), *PROZ* [OMIM:176895](protein Z deficiency [OMIM:614024])	—
15q11.2	—	*NIPA1* [OMIM:608145] (spastic paraplegia 6, autosomal dominant [OMIM:600363]), *MKRN3* [OMIM:603856] (precocious puberty, central, 2 [OMIM:615346]), *MAGEL2* [OMIM:605283] (Prader-Willi-like syndrome [OMIM:615547]), *NDN* [OMIM:602117] (Prader-Willi syndrome [OMIM:176270]), *SNRPN* [OMIM:182279] (Prader-Willi syndrome [OMIM:176270])
15q11.2	—	*MKRN3* [OMIM:603856] (precocious puberty, central, 2 [OMIM:615346]), *MAGEL2* [OMIM:605283] (Schaaf-Yang syndrome [OMIM:615547]), *NDN* [OMIM:602117] (Prader-Willi syndrome [OMIM:176270]), *SNRPN* [OMIM:182279] (Prader-Willi syndrome [OMIM:176270])
15q13.1q14	*HERC2* [OMIM:605837] (mental retardation, autosomal recessive 38 [OMIM:615516],	*SLC12A6* [OMIM:604878] (agenesis of the corpus callosum

	skin/hair/eye pigmentation 1, blond/brown hair [OMIM:227220], skin/hair/eye pigmentation 1, blue/nonblue eyes [OMIM:227220])	with peripheral neuropathy [OMIM:218000]), *NOP10* [OMIM:606471] (dyskeratosis congenita, autosomal recessive 1 [OMIM:606471]), ACTC1 [OMIM:102540] (atrial septal defect 5 [OMIM:612794], cardiomyopathy, dilated, 1R [OMIM:613424], cardiomyopathy, familial hypertrophic, 11 [OMIM:612098], left ventricular noncompaction 4 [OMIM:613424])
16p11.2q12.1	—	*VPS35* [OMIM:601501] (Parkinson disease 17 [OMIM:614203]), ORC6 [OMIM:607213] (Meier-Gorlin syndrome 3 [OMIM:613803]), *GPT2* [OMIM:138210] (mental retardation, autosomal recessive 49 [OMIM:616281]), *PHKB* [OMIM:172490] (phosphorylase kinase deficiency of liver and muscle, autosomal recessive [OMIM:261750]), *ZNF423* [OMIM:604557] (Joubert syndrome 19 [OMIM:614844], nephronophthisis 14 [OMIM:614844]), *NOD2* [OMIM:605956] (Blau syndrome [OMIM:186580], sarcoidosis, early-onset [OMIM:609464], inflammatory bowel disease 1 [OMIM:266600], psoriatic arthritis, susceptibility to [OMIM:607507]), *CYLD* [OMIM:605018] (Brooke-Spiegler syndrome [OMIM:605041]; cylindromatosis, familial [OMIM:605018], trichoepithelioma, multiple familial, 1 [OMIM:601606])
16q22.3q23.3	*FA2H* [OMIM:611026] (spastic paraplegia 35, autosomal recessive [OMIM:612319]), *CHST6* [OMIM:605294] (macular corneal dystrophy [OMIM:217800]), *TMEM231* [OMIM:614949] (Joubert syndrome 20 [OMIM:614970], Meckel syndrome 11 [OMIM:615397]), *KARS* [OMIM:601421] (Charcot-Marie-Tooth disease, recessive intermediate, B [OMIM:613641], deafness,	*WWOX* [OMIM:605131] (epileptic encephalopathy, early infantile, 28 [OMIM:616211], esophageal squamous cell carcinoma, somatic [OMIM:133239], spinocrebellar ataxia, autosomal recessive 12 [OMIM: 614322]), *MAF* [OMIM:177075] (Ayme-Gripp

	autosomal recessive 89 [OMIM:613916]), *ADAMTS18* [OMIM:607512] (microcornea, myopic chorioretinal atrophy, and telecanthus [OMIM:615458])	syndrome [OMIM:601088], cataract 21, multiple types [OMIM:610202]), *GCSH* [OMIM:238330] (glycine encephalopathy [OMIM:605899])
16q24.2q24.3	*FBXO31* [OMIM:609102] (?mental retardation, autosomal recessive 45 [OMIM:615979]), *JPH3* [OMIM:605268] (Huntington disease-like 2 [OMIM:606438]), *CA5A* [OMIM:114761] (hyperammonemia due to carbonic anhydrase VA deficiency [OMIM:615751]), *ZNF469* [OMIM:612078](Brittle cornea syndrome 1 [OMIM:229200]), *CYBA* [OMIM:608508](chronic granulomatous disease, autosomal, due to deficiency of CYBA [OMIM:233690]), *MVD* [OMIM:603236](porokeratosis 7, multiple types [OMIM:614714]), *PIEZO1* [OMIM:611184] (dehydrated hereditary stomatocytosis with or without pseudohyperkalemia and/or perinatal edema [OMIM:194380], lymphatic malformation 6 [OMIM:616843]), *CDT1* [OMIM:605525] (Meier-Gorlin syndrome 4 [OMIM:613804]), *APRT* [OMIM:102600](adenine phosphoribosyltransferase deficiency [OMIM:614723]), *GALNS* [OMIM:612222](mucopolysaccharidosis IVA [OMIM:253000]), *ACSF3* [OMIM:614245] (combined malonic and methylmalonic aciduria [OMIM:614265]), *CDH15* [OMIM:114019](mental retardation, autosomal dominant 3 [OMIM:612580]), *ANKRD11* [OMIM:611192](KBG syndrome [OMIM:148050]), *SPG7* [OMIM:602783](spastic paraplegia 7, autosomal recessive [OMIM:607259])	—
17p13.3p13.2	*WDR81* [OMIM:614218](cerebellar ataxia, mental retardation, and dysequilibrium syndrome 2 [OMIM:610185]), *SERPINF2* [OMIM:613168] (alpha-2-plasmin inhibitor deficiency [OMIM:262850]), *SERPINF1* [OMIM:172860](osteogenesis imperfecta, type VI [OMIM:613982]), *PAFAH1B1* [OMIM:601545](lissencephaly 1 [OMIM:607432], subcortical laminar heterotopia [OMIM:607432]), *ASPA* [OMIM:608034] (Canavan disease [OMIM:271900]), *TRPV3* [OMIM:607066] (palmoplantar keratoderma,	

	nonepidermolytic, focal 2 [OMIM:616400], Olmsted syndrome [OMIM:614594]), *TRPV1* [OMIM:602076], *CTNS* [OMIM:606272](cystinosis, atypical nephropathic [OMIM:219800], cystinosis, late-onset juvenile or adolescent nephropathic [OMIM:219900], cystinosis, nephropathic [OMIM:219800], cystinosis, ocular nonnephropathic [OMIM:219750]), *P2RX1* [OMIM:600845](bleeding disorder, platelet-type [OMIM:609821], bleeding disorder due to P2RX1 defect, somatic [OMIM:609821])	
17p13.1p11.2	*MYH8* [OMIM:160741] (Carney complex variant [OMIM:608837], Trismus-pseudocamptodactyly syndrome [OMIM:158300]), *MYH2* [OMIM:160740] (proximal myopathy and ophthalmoplegia [OMIM:605637]), *MYH3* [OMIM:160720] (arthrogryposis, distal, type 2A [OMIM:193700], arthrogryposis, distal, type 2B [OMIM:601680]), *SCO1* [OMIM:603644] (hepatic failure, early onset, and neurologic disorder), *COX10* [OMIM:602125] (Leigh syndrome due to mitochondrial *COX4* deficiency [OMIM:256000], mitochondrial complex IV deficiency [OMIM:220110])	TTC19 [OMIM:613814] (mitochondrial complex III deficiency, nuclear type 2 [OMIM:615157]), CPI [OMIM:119540] (cleft palate, isolated [OMIM:119540]), TNFRSF13B [OMIM:604907] (immunodeficiency, common variable, 2 [OMIM:240500], immunoglobulin A deficiency 2 [OMIM:609529])
17q25.3	*CCDC40* [OMIM:613799] (ciliary dyskinesia, primary, 15 [OMIM:613808]), *GAA* [OMIM:606800] (glycogen storage disease II [OMIM:232300]), *EIF4A3* [OMIM:608546] (Robin sequence with cleft mandible and limb anomalies [OMIM:268305]), *CARD14* [OMIM:607211] (pityriasis rubra pilaris [OMIM:173200], psoriasis 2 [OMIM:602723]), *SGSH* [OMIM:605270] (mucopolysaccharidosis type IIIA (Sanfilippo A) [OMIM:252900]), *ACTG1* [OMIM:102560] (Baraitser-Winter syndrome 2 [OMIM:614583], deafness, autosomal dominant 20/26 [OMIM:604717]), *FSCN2* [OMIM:607643] (retinitis pigmentosa 30 [OMIM:607921]), *PDE6G* [OMIM:180073] (retinitis pigmentosa 57 [OMIM:613582]), *GCGR* [OMIM:138033]({diabetes mellitus, noninsulin-dependent} [OMIM:125853]), *P4HB* [OMIM:176790] (Cole-Carpenter syndrome 1 [OMIM:112240]), *ARHGDIA* [OMIM:601925] (nephrotic syndrome, type 8 [OMIM:615244]), *PYCR1* [OMIM:179035] (cutis laxa, autosomal recessive, type IIB [OMIM:612940], cutis laxa, autosomal	

	recessive, type IIIB [OMIM:614438]), *ASPSCR1* [OMIM:606236] (alveolar soft-part sarcoma [OMIM:606243]), *DCXR* [OMIM:608347] ([pentosuria] [OMIM:260800]) *CSNK1D* [OMIM:600864] (advanced sleep-phase syndrome, familial, 2 [OMIM:615224])	
22q11.1q11.21	*IL17RA* [OMIM:605461] (candidiasis, familial, 5, autosomal recessive [OMIM:613953]), *CECR2* [OMIM:607576], *CECR1* [OMIM:607575] (Sneddon syndrome [OMIM:182410], polyarteritis nodosa, childhood-onset [OMIM:615688]), *PEX26* [OMIM:608666] (peroxisome biogenesis disorder 7A (Zellweger) [OMIM:614872], peroxisome biogenesis disorder 7B [OMIM:614873]), *TUBA8* [OMIM:605742] (polymicrogyria with optic nerve hypoplasia [OMIM:613180])	SCARF2 [OMIM:613619] (Van den Ende-Gupta syndrome [OMIM:600920]), PI4KA [OMIM:600286] (polymicrogyria, perisylvian, with cerebellar hypoplasia and arthrogryposis [OMIM:616531]), HCF2 [OMIM:142360] (thrombophilia due to heparin cofactor II deficiency [OMIM:612356]), SNAP29 [OMIM:604202] (cerebral dysgenesis, neuropathy, ichthyosis, and palmoplantar keratoderma syndrome [OMIM:609528]), CRKL [OMIM:602007], LZTR1 [OMIM:600574] (Noonan syndrome 10 [OMIM:616564], schwannomatosis-2, susceptibility to [OMIM:615670])
22q11.1q11.22	*IL17RA* [OMIM:605461] (candidiasis, familial, 5, autosomal recessive [OMIM:613953]), *CECR1* [OMIM:607575] (Sneddon syndrome recessive [OMIM:182410], polyarteritis nodosa, childhood-onset [OMIM:615688]), *PEX26* [OMIM:608666] (peroxisome biogenesis disorder 7A (Zellweger) [OMIM:614872], eroxisome biogenesis disorder 7B [OMIM:614873]), *TUBA8* [OMIM:605742] (polymicrogyria with optic nerve hypoplasia [OMIM:613180]), *PRODH* [OMIM:606810] (hyperprolinemia, type I [OMIM:239500]), schizophrenia, susceptibility to, 4 [OMIM:600850]),	RSPH14 [OMIM:605663], (Noonan syndrome 10 [OMIM:616564], schwannomatosis-2, susceptibility to [OMIM:615670], tetralogy of fallot [OMIM:187500], velocardiofacial syndrome [OMIM:192430])
22q11.1q11.23	*XKR3* [OMIM:611674], *IL17RA* [OMIM:605461] (immunodeficiency 51 [OMIM:613953]), *CECR1* [OMIM:607575] (?Sneddon syndrome [OMIM:182410], polyarteritis nodosa, childhood-onset [OMIM:615688]), *CECR2* [OMIM:607576], *ATP6V1E1* [OMIM:108746] (cutis laxa, autosomal recessive, type IIC [OMIM:617402]), *PEX26* [OMIM:608666]	—

	(peroxisome biogenesis disorder 7A (Zellweger) [OMIM:614872], peroxisome biogenesis disorder 7B [OMIM: 614873]), *TUBA8* [OMIM:605742] (cortical dysplasia, complex, with other brain malformations 8 [OMIM:613180])	
Xp22.32p22.2	*KAL1* [OMIM:300836] (hypogonadotropic hypogonadism 1 with or without anosmia (Kallmann syndrome 1) [OMIM:308700]), *TBL1X* [OMIM:300196] (Hypothyroidism, congenital, nongoitrous, 8) [OMIM:301033]	NLGN4X [OMIM:300427] (mental retardation, X-linked [OMIM:300495], Asperger syndrome susceptibility, X-linked 2 [OMIM:300497], autism susceptibility, X-linked 2 [OMIM:300495]),
Xp21.1p11.4	*DMD* [OMIM:300377](Becker muscular dystrophy [OMIM:300376], cardiomyopathy, dilated, 3B [OMIM:302045], Duchenne muscular dystrophy [OMIM:310200])	XK [OMIM:314850](McLeod syndrome with or without chronic granulomatous disease [OMIM:300842]), CYBB [OMIM:300481](chronic granulomatous disease, X-linked [OMIM:306400], immunodeficiency 34, mycobacteriosis, X-linked [OMIM:300645]), RPGR [OMIM:312610](cone-rod dystrophy, X-linked, 1 [OMIM: 304020], macular degeneration, X-linked atrophic [OMIM: 300834], retinitis pigmentosa 3 [OMIM: 300029], retinitis pigmentosa, X-linked, and sinorespiratory infections, with or without deafness [OMIM: 300455]), OTC [OMIM:300461](ornithine transcarbamylase deficiency [OMIM: 311250]), TSPAN7 [OMIM:300096] (mental retardation, X-linked 58 [OMIM: 300210])
